# Interleukin-17A Gene Expression in Morbidly Obese Women

**DOI:** 10.3390/ijms160817469

**Published:** 2015-07-30

**Authors:** Fernando Zapata-Gonzalez, Teresa Auguet, Gemma Aragonès, Esther Guiu-Jurado, Alba Berlanga, Salomé Martinez, Andreu Martí, Fátima Sabench, Mercé Hernandez, Carmen Aguilar, Joan Josep Sirvent, Rosa Jorba, Daniel Del Castillo, Cristóbal Richart

**Affiliations:** 1 Grup de Recerca GEMMAIR (AGAUR)—Medicina Aplicada, Departament de Medicina i Cirurgia, Institut d’Investigació Sanitària Pere Virgili (IISPV), Universitat Rovira i Virgili (URV), 43007 Tarragona, Spain; E-Mails: fernandoa.zapata@gmail.com (F.Z.-G.); tauguet.hj23.ics@gencat.cat (T.A.); gemma.aragones@iispv.cat (G.A.); esther.guiu@urv.cat (E.G.-J.); alba.berlanga@urv.cat (A.B.); caguilar.hj23.ics@gencat.cat (C.A.); 2 Servei Medicina Interna, Hospital Universitari Joan XXIII Tarragona, Mallafré Guasch, 4, 43007 Tarragona, Spain; E-Mail: andreumano@gmail.com; 3 Servei Anatomia Patològica, Hospital Universitari Joan XXIII Tarragona, Mallafré Guasch, 4, 43007 Tarragona, Spain; E-Mails: mgonzalez.hj23.ics@gencat.cat (S.M.); jsirvent.hj23.ics@gencat.cat (J.J.S.); 4 Servei de Cirurgia, Hospital Sant Joan de Reus, Departament de Medicina i Cirurgia, IISPV, Universitat Rovira i Virgili (URV), Avinguda Doctor Josep Laporte, 2, 43204 Tarragona, Spain; E-Mails: fatima.sabench@urv.cat (F.S.); mhernandezg@grupsagessa.com (M.H.); ddelcastillo@grupsagessa.com (D.D.C.); 5 Servei de Cirurgia, Hospital Universitari Joan XXIII Tarragona, Mallafré Guasch, 4, 43007 Tarragona, Spain; E-Mail: rjorba.hj23@gencat.cat

**Keywords:** IL-17A, morbid obesity, adipo/cytokine, visceral adipose tissue

## Abstract

Data from recent studies conducted in rodent models and humans suggest that interleukin-17A (IL-17A) plays a role in the induction of inflammation in adipose tissue during obesity. The aim of this study was to assess the gene expression of IL-17A in adipose tissue of morbidly obese patients. We used RT-PCR to evaluate the expression of IL-17A and several adipo/cytokines in the visceral adipose tissue (VAT) and subcutaneous adipose tissue (SAT) of 10 normal-weight control women (BMI < 25 kg/m^2^) and 30 morbidly obese women (MO, BMI > 40 kg/m^2^). We measured serum levels of IL-17A and adipo/cytokines in MO and normal weight women. IL-17A expression was significantly higher in VAT than in SAT in MO patients (*p* = 0.0127). It was very low in normal-weight controls in both VAT and SAT tissues. We found positive correlations between IL-17A and IL-6, lipocalin-2 and resistin in VAT of MO patients. The circulating level of IL-17A was higher in the normal-weight group than the MO patients (*p* = 0.032), and it was significantly related to adiponectin and TNFRII levels. In conclusion, IL-17A expression in VAT is increased in morbidly obese women, which suggests a link between obesity and innate immunity in low-grade chronic inflammation in morbidly obese women.

## 1. Introduction

Nowadays, obesity is perceived not only as an a esthetic drawback, but also as a serious, almost pandemic, health problem associated with an increased risk of developing such diseases as type 2 diabetes mellitus, metabolic syndrome, cardiovascular disease, cancer and autoimmune diseases [1]. Nutrient excess and adiposity activate several metabolic pathways implicated in the development of insulin resistance, including inflammatory signaling, lipotoxicity, aberrant adipokine secretion, adipose tissue hypoxia, endoplasmic reticulum stress and mitochondrial dysfunction [2,3,4,5,6,7,8]. Several of these metabolic processes can converge in the development of metabolic inflammation. The first indication that inflammatory mediators are associated with obesity was the discovery of the increased expression of the pro-inflammatory cytokine tumor necrosis factor (TNF) α in adipose tissue of obese mice almost two decades ago [9]. Subsequent studies have shown that changes in inflammatory signaling by adipocytes and infiltration of adipose tissue by immune cells are key features of obesity-induced insulin resistance and associated metabolic disease in animal models and humans [10,11,12]. In obese mice, both adipocytes and macrophages (and potentially other cell types) residing in adipose tissue secrete a number of cytokines, including TNFα, interleukin (IL)-6, IL-1β and migration inhibitory factor [11]. Increased expression of inflammatory mediators has also been observed in the visceral fat of obese humans and mice [13]. In this regard, obesity leads to a state of low-grade chronic inflammation in adipose tissues, with increased adipose tissue macrophage infiltration [14]. In addition to macrophages, other immune cells, including mediators of both innate and adaptive immune responses, also localize to adipose tissue in obesity [15]. Neutrophils, mast cells, natural killer T cells and lymphocytes have all been observed in white adipose tissue in response to a high-fat diet (HFD) or in conditions of obesity [16,17,18,19]. However, the precise point at which this infiltration occurs during disease progression and their pro-inflammatory cytokine production in the pathogenesis of metabolic dysfunction in obese people remains to be determined.

Unlike “classic” pro-inflammatory mediators, for example TNF-α, IL-6 and C-reactive protein, T cell-derived cytokines, such as IL-17A, have not been extensively investigated in obesity. Mainly induced by monocyte/dendritic cell-derived IL-23, IL-17A has been implicated not only in host defense, but also in the pathogenesis of several autoimmune disorders and cancer [20]. It has recently been suggested that IL-17A plays a role in the induction of inflammation in adipose tissue during obesity, glucose homeostasis and adipogenesis [21]. Despite the fact that it has been reported that inflammatory cytokines, such as IL-17A regulates the differentiation of adipocytes and their capacity to secrete adipo/cytokines, the relationship between IL-17A and other adipo/cytokines is still unknown. Moreover, some studies have shown that serum IL-17A is upregulated in obese human patients [22]. Obesity is also positively correlated with increased IL-17A expression and increased severity of inflammation in IL-17A-dependent mouse models [23]. Although these studies suggest a link between obesity and IL-17, their pro-inflammatory cytokine role in the metabolic dysfunction of obese people is not completely understood.

On the basis of the above data and to better understand the mechanisms causing or maintaining the dysfunction of adipose tissue, the aim of the present study was to assess IL-17A in low-grade chronic inflammation due to obesity by: (1) evaluating the expression of IL-17A and several adipo/cytokines in both visceral (VAT) and subcutaneous adipose tissue (SAT); (2) analyzing the circulating levels of IL-17A and other adipo/cytokines from morbidly obese patients and normal-weight healthy subjects.

## 2. Results

### 2.1. Baseline Characteristics of Subjects

Table 1 shows the general characteristics, biochemical and metabolic measurements of the population studied. We classified the patients into two groups according to their body mass index (BMI): normal-weight patients (BMI < 25 kg/m^2^), who acted as controls and morbidly obese women (MO; BMI > 40 kg/m^2^). The two groups were comparable in terms of age (*p* = 0.442). As expected, biochemical analyses indicated that MO patients had significantly higher levels of fasting glucose, insulin, homeostasis model assessment of insulin resistance (HOMA2-IR), glycated hemoglobin (HbA1c), systolic blood pressure (SBP) and diastolic blood pressure (DBP) (*p* < 0.05) than normal-weight subjects. There was no difference in lipid profile, because the morbidly obese women were taking lipid-lowering drugs.

**Table 1:** General baseline characteristics and metabolic variables of the cohort studied: normal-weight control women and morbidly obese women.

Variables	Normal-Weight Control (*n* = 10)	Morbidly Obese (*n* = 30)
	Mean ± SD	Mean ± SD
Age (years)	43.70 ± 12.35	47.00 ± 7.35
Weight (kg)	56.30 ± 8.64	123.69 ± 13.18 *
WC (cm)	68.60 ± 10.5	135.16 ± 11.32 *
BMI (kg/m_2_)	22.61 ± 1.91	47.21 ± 5.4 *
SBP (mmHg)	119.88 ± 13.08	139.10 ± 14.78 *
DBP (mmHg)	67.55 ± 7.57	80.26 ± 14.76 *
Glucose (mg/dL)	88.11 ± 9.63	115.5 ± 26.12 *
Insulin (mU/L)	7.93 ± 5.86	20.26 ± 13.99 *
HbA1c (%)	4.55 ± 0.28	5.7 ± 1.22 *
HOMA2-IR	1.07 ± 0.83	2.69 ± 1.77 *
Cholesterol (mg/dL)	172.68 ± 25.91	179.39 ± 35.22
HDL-C (mg/dL)	54.66 ± 17.85	41.26 ± 7.52
LDL-C (mg/dL)	93.77 ± 26.74	106.13 ± 28.64
Triglycerides (mg/dL)	121 ± 79.32	159.6 ± 53.25
**Adipo/cytokine circulating levels**		
Adiponectin (µg/mL)	11.48 ± 6.13	6.77 ± 2.84 *
IL-6 (pg/mL)	1.78 ± 1.55	2.95 ± 1.56
Lipocalin-2 (ng/mL)	63.53 ± 28.33	82.26 ± 29.85
Resistin (ng/mL)	3.61 ± 1.31	4.52 ± 1.63
TNFRII (ng/mL)	3.09 ± 1.51	5.19 ± 2.37 *

WC, waist circumference; BMI, body mass index; SBP, systolic blood pressure; DBP, diastolic blood pressure; HbA1c, glycosylated hemoglobin; HOMA2-IR, homeostasis model assessment of insulin resistance; HDL-C, high-density lipoprotein; LDL-C, low-density lipoprotein. * Significant differences compared to normal-weight controls (*p* < 0.05). Data are expressed as the mean ± SD.

### 2.2. IL-17A and Adipo/Cytokine mRNA Expression in Adipose Tissue

**Figure 1:**
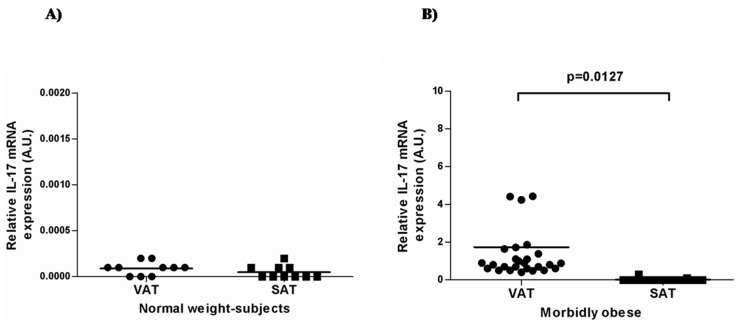
IL-17A mRNA expression in visceral and subcutaneous adipose tissues in normal-weight subjects (**A**) and morbidly obese women (**B**). AU: arbitrary units × 10^4^; VAT: visceral adipose tissue; SAT: subcutaneous adipose tissue. Student’s *t*-test was used to compare the gene expression in the two groups. Results are shown as the mean ± SD. *p* < 0.05 is considered statistically significant.

First, we analyzed the expression of IL-17A in visceral and subcutaneous adipose tissue in both normal-weight patients and MO. IL-17A mRNA expression was very low (almost undetectable) in normal-weight controls in both VAT and SAT tissues (Figure 1A), whereas in MO patients, IL-17A expression was significantly higher in VAT than in SAT (*p* = 0.0127, Figure 1B).

In order to determine whether IL-17A can induce pro-inflammatory cytokine IL-6 secretion, we also evaluated IL-6 mRNA expression in both human adipose tissues in normal-weight subjects and MO. We found that IL-6 expression was higher in the VAT and SAT of MO women than in the normal-weight group (Table 2, *p* < 0.001). In the MO group, there were no differences between VAT and SAT tissues.

**Table 2:** Adipo/cytokines gene expression in morbidly obese patients and normal-weight subjects. VAT, visceral adipose tissue; SAT, subcutaneous adipose tissue.

Gene Expression	Normal-Weight Control (*n* = 10)		Morbidly Obese (*n* = 30)	
	VAT	SAT	VAT	SAT
	Mean ± SD	Mean ± SD	Mean ± SD	Mean ± SD
IL-6	2.38 ± 1.52	17. 07 ± 11.66	61.09 ± 14.13 *	86.40 ± 28.71 *
Adiponectin	0.77 ± 0.32	0.58 ± 0.30	0.53 ± 0.27	0.29 ± 0.17 *
Lipocalin-2	0.006 ± 0.003	0.029 ± 0.02	0.02 ± 0.01	0.064 ± 0.03
TNFα	0.039 ± 0.02	0.076 ± 0.06	0.26 ± 0.20 *	0.043 ± 0.02
Resistin	0.008 ± 0.004	0.016 ± 0.005	0.030 ± 0.02 *	0.030 ± 0.02

Student’s *t*-test was used to compare the gene expression in the two groups. * Indicates significant differences with respect to the normal-weight group (*p* < 0.05). The mRNA expression for each gene and sample was calculated using the recommended 2^−Δ*C*t^ method. Data are expressed as the mean ± SD.

**Figure 2:**
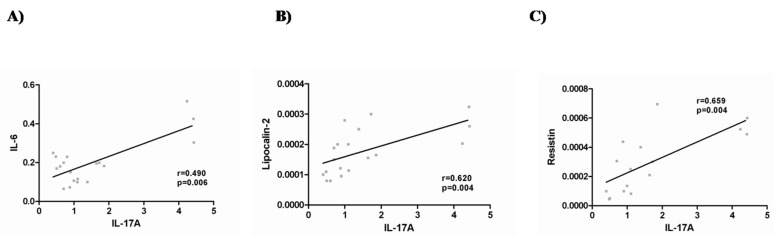
Correlation between mRNA expression of IL-17A and IL-6 (**A**), lipocalin-2 (**B**) and resistin (**C**) in visceral adipose tissue of morbidly obese women. The strength of association between variables was calculated using Spearman’s *r* correlation test. *p* < 0.05 is considered statistically significant.

As far as adipo/cytokine expression was concerned, we found that resistin and TNFα were increased in the VAT of MO patients in comparison to normal-weight controls, whereas adiponectin was higher in the SAT control group (Table 2). In the MO group, we found positive correlations between IL-17A expression and IL-6, lipocalin-2 and resistin expressions in VAT tissue (Figure 2).

### 2.3. IL-17A and Adipo/Cytokine Circulating Levels

**Figure 3:**
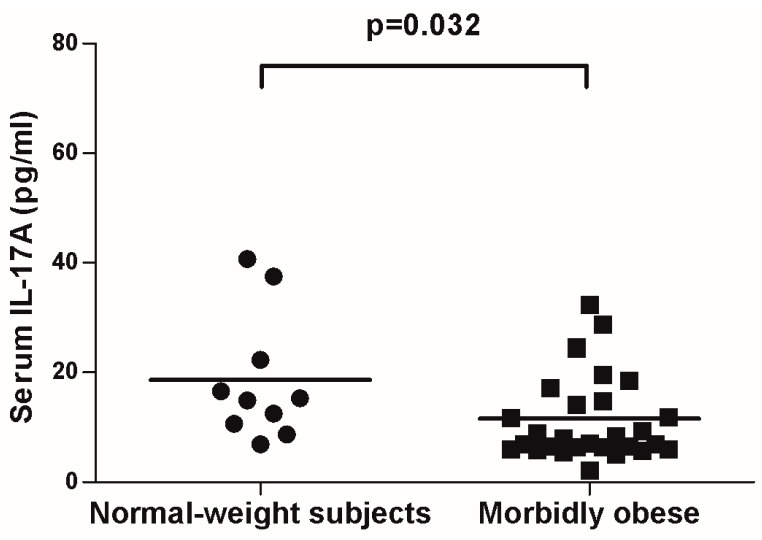
Circulating levels of IL-17A in normal-weight subjects (BMI < 25) and morbidly obese women (BMI > 40). Student’s *t*-test was used to compare the gene expression in the two groups. Results are shown as the mean ± SD. *p* < 0.05 are considered statistically significant.

In order to study whether the differences observed in mRNA expression were only a local effect or if they were also reflected in serum, we measured circulating levels in both groups. Figure 3 shows that the IL-17A serum concentration was higher in the normal-weight group than in the MO patients (*p* = 0.032).

We also compared the circulating levels of adipo/cytokines in the MO group with controls (Table 1), and analyzed their association with serum IL-17A levels. As expected, we found that adiponectin circulating levels were higher in the normal-weight group than in the MO patients (*p* = 0.003). In contrast, we observed that TNFRII levels were significantly higher in MO patients than in normal-weight subjects (*p* = 0.019).

Then, we assessed the relationship between IL-17A and adipo/cytokine levels in serum. In the whole population studied, we found that IL-17A was positively related to adiponectin and inversely to TNFRII levels (*r* = 0.367, *p* = 0.028; *r* = −0.479, *p* = 0.004; respectively). In MO patients, these correlations were significant (*r* = 0.503, *p* = 0.007; *r* = −0.456, *p* = 0.019; respectively).

## 3. Discussion

Recent studies showed the potential implication of IL-17A in human obesity-linked inflammation and co-morbidities [24,25,26,27]. Thus, we analyzed the gene expression of IL-17A and several adipo/cytokines in VAT and SAT samples from morbidly obese and normal-weight women. We also evaluated their circulating levels in both groups. We demonstrated that IL-17A mRNA expression was almost undetectable in normal-weight controls in both VAT and SAT tissues. However, IL-17A expression was significantly higher in VAT than in SAT in MO women. Paradoxically, we found that IL-17A serum levels were higher in the normal-weight women than in the MO women.

In many senses, obesity is considered to be an inflammatory predisposition. For instance, low levels of chronic inflammation and macrophage infiltration into adipose tissue are associated with obese conditions [28]. Obesity is also noted to predispose to several autoimmune disorders [29,30,31]. Although the mechanisms connecting both phenomena remain elusive, important recent evidence has suggested that IL-17A is a key element in these processes. Not only can it regulate adipogenesis and glucose homeostasis in murine obesity, but it is also associated with chronic inflammation processes and autoimmune disorders [20,21]. Recent studies have reported that CD4+ T cells are increased in adipose tissue of overweight and obese patients [26,32]. In view of these previous data, we have been able to confirm that IL-17A is present in the adipose tissue of MO women. According to our results, Bertola *et al.* found that expression of IL-17A in the stromal vascular fraction from adipose tissue was increased in overweight and obese patients compared to lean subjects [32]. Surprisingly, we found that IL-17A expression was much lower than that of the housekeeping gene, especially in subcutaneous adipose tissue. This can be partially explained, at least in humans, by self-regulatory mechanisms that limit the expansion of TH17 cells (impaired IL-2 production and arrest of cell cycle progression) and the high transience of these cells, which rapidly shift to a Th1 profile [33]. Some recent studies have shown that obesity is directly related to IL-17A expression and increased severity of inflammation in IL-17A-dependent mouse models [23]. In rodent models, Zuñiga *et al.* [21] showed that IL-17 is expressed by γδ T cells in white adipose tissue. Interestingly, we observed that IL-17A expression is greater in VAT than in SAT. In this regard, fat stored in visceral adipose depots makes obese individuals more prone to metabolic complications than fat distributed subcutaneously [34]. Another important fact to note is that IL-17A mRNA expression was almost undetectable in normal-weight controls in both VAT and SAT tissues, which may be due to the lack of adipose tissue inflammation in this group of subjects [35].

It has been widely shown that IL-6 expands the TH17 fraction in obesity while being concurrently induced by IL-17A itself [36]. Our findings showed a positive correlation between IL-17A and IL-6 expression in the VAT of MO patients. IL-17A could be related to low-grade inflammation in obese patients, as it is capable of inducing other pro-inflammatory mediators, such as IL-6, and inducing neutrophil chemotaxis [36,37]. In this regard, it has been reported that IL-17A can stimulate the production of IL-6 by activating such signaling pathways as NF-κB, Stat3 or PI3K/Akt [38]. Thus, IL-17 is a cytokine-inducing cytokine, and the interaction between IL-17A and IL-6 can increase the levels of both cytokines. It is also known that IL-6 is required for the differentiation of naive CD4 T cells into the Th17 lineage [39,40] and is a major downstream gene target of IL-17A [41,42].

Furthermore, in VAT tissue, we found positive correlations between IL-17A and lipocalin-2, two adipose tissue-derived cytokines, and resistin expression. Lipocalin-2 seems to act as a dual molecule with regard to inflammation in obesity [43,44,45]. Increased adipose tissue expression of resistin has been previously described in obesity [46]. These and many other adipo/cytokines play a physiological and pro-inflammatory role in metabolism and are involved in the development of obesity, inflammation and auto-immune disorders [47].

As far as circulating levels are concerned, IL-17A is mainly secreted by the activated CD4+ and CD8+ T lymphocytes and has been classified as a pro-inflammatory cytokine because of its ability to induce the expression of many mediators of inflammation [48]. The morbidly obese women in our study had less IL-17 in the circulation than normal-weight controls. This finding did not coincide with that of a previous publication that reported increased IL-17A circulating levels in the plasma of obese women [22]. This inconsistency might stem from the dissimilarities in the populations studied in terms of age and obesity grade. It is known that aging is accompanied by a progressive increase in pro-inflammatory cytokines, including IL-17A [49]. In this sense, the cohort of Sumarac-Dumanovic *et al.* has an age of 35.0 ± 8.5 years [22]. However, our cohort of morbidly obese women was older. Regarding obesity grade, although it is not possible to determine the causality of the fact that the levels of IL-17A were decreased in serum of obese patients, the reported results suggest that in this type of extreme obesity, adipose tissue behaves in a differential way than expected [13]. We also observed that IL-17A circulating levels were positively related to adiponectin and negatively to TNFRII levels, as was also observed by Xuan *et al.* [50]. Taken together, these results do not clarify whether IL-17A acts as a pro-inflammatory molecule. Further studies of IL-17A circulating levels are needed to understand these controversial results.

Our study cohort enabled us to confirm the expression of IL-17A in adipose tissue of the morbidly obese without the interference of such confounding factors as gender or age. However, the results of our study cannot be extrapolated to other obesity groups or to men. Furthermore, our findings indicate that there may be a late adaptive process in this type of extreme obesity.

## 4. Materials and Methods

### 4.1. Subjects

The study was approved by the institutional review board “Comitè d’Ètica d’Investigació Clínica, Hospital Universitari de Tarragona Joan XXIII” (6proj2; 25 June 2009). All participants gave written informed consent for participation in medical research. We analyzed gene expression in paired samples of subcutaneous and visceral adipose tissue from 40 women of Western European descent: 10 normal weight (BMI < 25 kg/m^2^) and 30 morbidly obese (BMI > 40 kg/m^2^). Adipose tissue samples were obtained from morbidly obese women who underwent bariatric surgery by laparoscopic gastric bypass and from lean patients who underwent laparoscopic cholecystectomy for benign gall bladder disease or laparoscopic hiatal hernia repair. Subcutaneous adipose tissue biopsies were taken from the right hypochondriac region, and visceral adipose tissue biopsies were taken from the greater epiploon region. Samples were obtained by the same specialist in each surgical case. Morbidly obese women and controls were similar age. The weight of all subjects had been stable for at least 3 months before surgery. Patients who had an acute illness, acute or chronic inflammatory or infective diseases, or end-stage malignant disease were excluded. Menopausal women and women receiving contraceptive treatment were also excluded.

### 4.2. Biochemical Analyses

The anthropometric and metabolic characteristics of the subjects were determined. Body height and weight were measured with the patient standing shoeless and in light clothes. BMI was calculated as body weight divided by height squared (kg/m^2^). Laboratory studies included glucose, insulin, HbA1c, total cholesterol, high-density lipoprotein cholesterol (HDL-C), low-density lipoprotein cholesterol (LDL-C) and triglycerides, all of which were analyzed using a conventional automated analyzer. The homeostasis model assessment of insulin resistance (HOMA2-IR) was completed using the HOMA calculator version 2.2.2 [51].

### 4.3. RNA Isolation and Real-Time PCR

Total RNA was isolated from adipose tissues in accordance with the manufacturer’s protocol for the RNeasy midi kit (Qiagen, Barcelona, Spain) and was digested with DNase I (RNase-Free DNase set, Qiagen). First-strand cDNA was synthesized using an equal amount of total RNA with a High Capacity RNA-to-cDNA Kit (Applied Biosystems, Madrid, Spain). Real-time quantitative PCR was performed in a final volume of 20 µL, which contained 10 ng of reverse-transcribed cDNA, 10 µL of 2× TaqMan Fast Universal PCR Master Mix (Applied Biosystems) and 1 µL TaqMan Assay predesigned by Applied Biosystems for the detection of IL-17A, IL-6, resistin, lipocalin-2, adiponectin, TNF receptor (R) II and GAPDH, which was used as housekeeping gene. All reactions were performed in triplicate and were carried out in 96-well plates using the 7900HT Fast Real-Time PCR System (Applied Biosystems).The mRNA expression for each gene and sample was calculated using the recommended 2^−Δ*C*t^ method.

### 4.4. IL-17A and Adipo/Cytokine Serum Levels

Circulating levels of IL-17A (Enzo Life Sciences, Farmingdale, NY, USA) and several adipo/cytokines—IL-6 (Quantikine, R&D Systems, Minneapolis, MN, USA), adiponectin (EMD Millipore, St. Charles, MI, USA), resistin (BioVendor, Brno, Czech Republic), lipocalin-2 (R&D Systems, Minneapolis, MN, USA) and TNFRII (AssayPro, St. Charles, IL, USA)—were measured in duplicate using enzyme-linked immunosorbent assays (ELISA) following the manufacturer’s instructions. The IL-17A assay sensitivity was 0.201 pg/mL, and intra-assay and inter-assay coefficients of variation (CV) were 5.4% and 9.4%, respectively. IL-6 assay sensitivity was 0.039 pg/mL, and intra-assay and inter-assay CV were 7.4% and 7.8%, respectively. Adiponectin assay sensitivity was 0.2 ng/mL, and intra-assay and inter-assay CV were 3.4% and 5.7%, respectively. Resistin assay sensitivity was 0.012 ng/mL, and intra-assay and inter-assay CV were 5.9% and 7.6%, respectively. Lipocalin-2 assay sensitivity was 0.012 ng/mL, and intra-assay and inter-assay CV were 3.7% and 6.5%, respectively. Finally, the TNFRII assay sensitivity was 0.1 ng/mL and the inter-assay and intra-assay coefficients of variation were less than 3.2% and 3.3%, respectively.

### 4.5. Statistical Analysis

All of the values reported were analyzed using the SPSS/PCC statistical package for Windows (v.20.0; Chicago, IL, USA). Differences between groups were calculated using Student’s *t*-test. The strength of association between variables was calculated using Pearson’s method for parametric variables and Spearman’s *r* correlation test for non-parametric contrasts. *p*-values < 0.05 were considered to be statistically significant. Data are expressed as the mean ± SD.

## 5. Conclusions

In conclusion, IL-17A mRNA expression was almost undetectable in normal-weight controls in both VAT and SAT tissues. However, IL-17A expression in visceral adipose tissue is increased in morbidly obese women and was associated with IL-6, lipocalin-2 and resistin expression in VAT. These findings confirm that there is a link between obesity and innate immunity in low-grade chronic inflammation in morbidly obese women. Further studies of IL-17A circulating levels are needed to understand these controversial results.

## Abbreviations

BMI: body mass index
VAT: visceral adipose tissue
SAT: subcutaneous adipose tissue
MO: morbidly obese
IL-17A: interleukin-17A
IL-6: interleukin 6
HbA1c: glycosylated hemoglobin
HOMA2-IR: homeostasis model assessment of insulin resistance
HDL-C: high density lipoprotein
LDL-C: low density lipoprotein
